# Risk factors associated with intracranial hemorrhage in neonates with persistent pulmonary hypertension on ECMO

**DOI:** 10.1186/s40560-015-0071-x

**Published:** 2015-02-11

**Authors:** Sule Doymaz, Marcia Zinger, Todd Sweberg

**Affiliations:** Pediatric Critical Care Division, Cohen Children’s Medical Center of New York, New Hyde Park, NY 11040 USA; Department of Pediatrics and Division of Pediatric Intensive Care Unit, SUNY DOWNSTATE Medical Center, 450 Clarkson Ave, Brooklyn, NY 11203 USA

**Keywords:** Intracranial hemorrhage, Neonates, ECMO, Pulmonary, Hypertension

## Abstract

**Background:**

Up to 40% of infants with persistent pulmonary hypertension (PPHN) remains refractory to conventional therapies, and extracorporeal membrane oxygenation (ECMO) is offered as an effective support for this group. However, ECMO is a highly invasive and risky procedure with devastating complications such as intracranial hemorrhage (ICH). In this study, we aimed to determine the risk factors for ICH in infants with PPHN.

**Methods:**

A case-control study of patients admitted to the pediatric intensive care unit (PICU) with PPHN requiring ECMO support was conducted. The study was carried out at a 25-bed PICU in large urban tertiary care children’s hospital. A total number of 32 subjects were studied. Patients with and without ICH during ECMO were evaluated for activated clotting time (ACT), heparin dosing, platelet count, coagulation profile such as activated partial thromboplastin time (aPTT), prothrombin time (PT), international normalized ratio (INR), fibrinogen level, vital signs including heart rate and mean arterial pressure (MAP), transfusion history, gestational age, and severity of pre-ECMO illness as possible risk factors.

**Results:**

Low fibrinogen level (115 ± 13 mg/dl) and low platelet counts (37.4 ± 18.3 Thousand/μl) were associated with higher incidence of ICH (*p* = 0.009 and *p* = 0.005, respectively). Elevated MAP (69 ± 4.34 mmHg) was also noticed in ICH patients (*p* = 0.006).

**Conclusions:**

Results demonstrated that low fibrinogen level and low platelet count were associated with ICH in PPHN patients on ECMO. While on ECMO support, maintaining fibrinogen and platelet counts within normal ranges seems crucial to prevent ICH in PPHN patients. This is the first report identifying low fibrinogen level among the risk factors for ICH in infants with PPHN on ECMO support.

## Background

Extracorporeal membrane oxygenation (ECMO) is an advanced support for patients who fail to respond to conventional therapy [1]. With ECMO support, survival rate has been improved for many life-threatening conditions related to respiratory failure over the years [1,2]. The overall survival rate for neonates with respiratory failure have been treated with ECMO was reported to be 79.7%, and the survival rate for persistent pulmonary hypertension (PPHN) patients was reported as 79.4% [2].

Occurring in 1.9 in 1,000 live births, PPHN is a condition where there is a failure of normal circulatory transition after birth [3]. As a result, the normal drop in pulmonary vascular resistance (PVR) fails to take place after birth leading to hypoxemia [4]. Both non-invasive and invasive mechanical ventilation as well as inhaled nitric oxide are used to provide adequate tissue oxygenation until PVR drops and normal circulatory dynamics are established [5]. Despite improvements in these conventional therapies, up to 40% of infants with PPHN remain refractory to therapy. For this group of patients, ECMO has been offered as an effective therapy since its adoption in the 1980s [3,5].

Despite its success, ECMO remains a highly invasive and risky procedure. In the neonatal population, one of the most devastating complications is intracranial hemorrhage (ICH) [1,6]. The need for anticoagulation in combination with the immature neonatal brain parenchyma makes the risk of ICH particularly high in this population. In previous studies, intracranial complications were reported as 25% to 50% with a survival rate of 43% [6].

Tubings and membranes of ECMO circuits are risk factors for the activation of the coagulation system [7]. Unfractionated heparin (UFH) is used in to prevent clots in ECMO circuit and the complications related to clot formation in patients. It is crucial to monitor the level of anticoagulation therapy closely to prevent the complications such as ICH as well as to track biochemical parameters such as activated partial thromboplastin time (aPTT), activated clotting time (ACT), and anti-factor Xa (anti-Xa) activity [7].

Relying on a single method has drawbacks. For example, PTT is used to determine the deficiencies of various coagulation factors and to monitor the heparin treatment. Prolonged PTT could be caused by many factors including liver diseases, deficiencies in coagulation factors and vitamin K, disseminated intravascular coagulation, etc.

Activated coagulation time (ACT) test is another method that is used for monitoring heparin treatment and is used with fresh blood at the bedside. In experienced hands, ACT produces reliable results. However, pre-analytical conditions such as the presence of hemolysis and suboptimal specimens might adversely affect the outcome.

A chromogenic assay named anti-Xa is utilized in quantifying UFH level. Factors such as free hemoglobin, bilirubin, and triglycerides in plasma may decrease the sensitivity of anti-Xa test [7].

Taken together, there is a need for a better delineation of the factors causing bleeding complications during the ECMO support [1]. The objective of the present study was to investigate the occurrence of ICH and to identify risk factors in neonates with PPHN.

## Methods

We retrospectively reviewed charts of infants between 1997 and 2010 and included all infants with PPHN diagnoses who required ECMO support. Infants who received ECMO support for all other pathologies such as meconium aspiration syndrome, congenital diaphragmatic hernia, acute respiratory distress syndrome, sepsis/pneumonia, or cardiac pathology were excluded. The reason for the exclusions was to study a uniform group of subjects. The aim was to reach a better comparison between patients with the same pathology. Different pathologies may affect coagulation system differently such as sepsis where patients may be more prone to develop disseminated intravascular coagulation or to bleeding post cardiac surgery. It is hoped that the uniformity of the subject might empower the analysis and the conclusion drawn from the study.

Data collection included: demographic information on age, sex, birth weight, ethnicity, type of ECMO, and duration on ECMO. Hourly ACT levels and heparin infusion doses for the first 12 h of ECMO and maximum ACT levels and heparin infusion doses for 5 days on ECMO for both groups were also collected. Coagulation profile for a-PTT, prothrombin time (PT), international normalized ratio (INR), and fibrinogen and platelet levels were analyzed. In our unit, the anticoagulation protocol for ECMO dictates that unfractionated heparin be used and infusion is given directly to ECMO circuit via an infusion port located before the bladder. The heparin infusion rate is determined by patient’s weight (in kg) × desired unit/kg/h (concentration of drip: 50 units/ml).

We check ACT hourly for a target value of 200–225 s. Target ACT values were adopted from Extracorporeal Life Support Organization (ELSO) guidelines [8]. All ACT levels are obtained from the ACT port located on the pre-membrane oxygenator diamond. PTT is monitored every 6 h during ECMO. Our target PTT level is 80–110 s. If ACT level is greater than the target range, we decrease the heparin infusion by 5–15 units/h. If the ACT is 20–25 s below the lower end of the target level or PTT is <65 s, a heparin bolus of 20 units/kg is given and infusion is increased by 5–15 units/h. If PTT level is >140, 10 ml/kg of fresh frozen plasma (FFP) is given. Platelet levels are checked every 6 h to a target value of >100,000, if less; platelet transfusion of 10 ml/kg is given. Fibrinogen level is checked every 12 h to a target value of >100 mg/dL. If the level is lower than the target value, cryoprecipitate is given to the patient. Heparin drip is increased by 5–15 units/h during platelet infusion and reduced to previous rate once the platelet infusion is completed.

Data were also collected for heart rate, mean arterial pressure (MAP) changes as well as minimum and maximum pH, PCO_2_, PaO_2_, and lactate levels. All infants were placed on ECMO support through neck cannulation. Decision for VA ECMO was based on patient’s clinical status, persistent and significant acidosis, higher level of respiratory support and hypoxemia, or signs of poor end organ perfusion. The hypoxia is described as having oxygenation index ≥40 for ≥4 h or persistently high oxygenation index for more than 12 h or PaO_2_ ≤40 despite maximum medical management.

Management of ICH on ECMO in our institution: Patients are decannulated if the intracranial hemorrhage is associated with profound clinical deterioration. If they remain asymptomatic, we maintain the lowest ACT possible (180–200) and repeat head ultrasound (HUS) more frequently. If ICH progresses, decannulation is considered as soon as full mechanical ventilator support can be achieved even if the settings are very high.

All infants had HUS evaluation prior ECMO cannulation. Baseline head US evaluations were negative for ICH before initiation of ECMO support. Daily HUS study was obtained to assess intracranial bleeding during ECMO. All ICH cases were diagnosed by HUS, and no CT scan were performed. All patients in the study were placed on prophylactic antibiotics. There was no mortality in our study population.

For statistical analyses, two-sample *t* test and chi-square tests were used and the null hypothesis was rejected at a *p* ≤ 0.05. In all statistical analyses, Minitab^R^ (State College, PA 16801, USA) Program was utilized.

The study received approval from IRB of North Shore LIJ Health System.

## Results

A total number of 32 subjects were included in the study. The primary reason for initiation of ECMO in all patients was the persistent hypoxia unresponsive to conventional therapy. Eleven patients were identified to have ICH by routine daily ultrasound, and 21 patients were without ICH. Demographic data is shown in Table 1. Mean duration of ECMO in hours was not significantly longer in the ICH group compared to the non-ICH group (*p* = 0.680). All factors and variables were measured and compared before ICH occurrence on both groups. The data on the maximum ACT levels and heparin infusion doses for 120 h on ECMO are shown in Table 2. We presented hourly ACT levels and heparin infusion doses for the two groups during the first 12 h on ECMO and found no statistical significance between the two groups (Table 2).

**Table 1:** **Patients demographic data**

	**ICH**	**No ICH**	***p*** **values**
Total number of patients: 32	11 (34%)	21 (66%)	
Gestational age in weeks (mean ± SD)	38.1 ± 1.2	38.3 ± 1.8	0.743
Gender (F/M)	4/7	5/16	0.453
Weight in kg (mean ± SD)	3.3 ± 0.2	3.4 ± 0.6	0.602
Type of ECMO (VV/VA)	4/7	9/12	0.722
Duration of ECMO in hour (mean ± SD)	183 ± 14	164 ± 11	0.680

**Table 2:** Maximum daily ACT values and maximum heparin infusion doses for each day*

	**Group**	**Day 1**	**Day 2**	**Day 3**	**Day 4**	**Day 5**
ACT in seconds (mean ± SD)	ICH	318.3 ± 54.8	260.8 ± 57.0	259.3 ± 62.2	264.8 ± 58.5	271.4 ± 57.3
	No ICH	313.7 ± 61.2	259.3 ± 29.0	252.2 ± 42.1	257.0 ± 33.2	255.6 ± 45.9
Max. heparin (mean ± SD)	ICH	32.1 ± 11.9	34.0 ± 11.4	40.2 ± 16.3	39.8 ± 11.3	37.8 ± 12.6
	No ICH	36.2 ± 11.8	37.6 ± 9.08	39.95 ± 7.93	43.5 ± 12.5	38.4 ± 13.9

*p values for maximum daily ACT values and maximum heparin infusion doses for each day were not statistically significant.

However, comparison of coagulation profiles indicated that the mean minimum platelet count and fibrinogen levels were significantly lower in the ICH group as demonstrated in Table 3 (*p* = 0.005 and 0.009, respectively). We found no statistical difference between a PTT, PT and INR values between the groups (Table 3). Similarly, mean volumes of platelet, fresh frozen plasma and cryoprecipitate transfusions for both groups were not significantly different between two groups (Table 3). Mean arterial pressure measurements were higher in the ICH group compared to the non-ICH group (69.36 ± 4.34 vs. 64.19 ± 4.86 mmHg with a *p* = 0.006) (Table 4). Minimum and maximum heart rate measurements were not statistically different between the two groups (Table 4). Similarly, the minimum pH values, maximum lactate levels, highest PaCO_2_, and lowest PaO_2_ in two groups did not differ significantly (Table 4).

**Table 3:** **Data related to coagulation profiles**

	**ICH ** **(mean ± ** **SD)**	**No ICH ** **(mean ± ** **SD)**	***p*** **values**
Min. platelet (Thousand/μl)	37.4 ± 18.3	60.4 ± 23.3	*0.005*
Max. platelet (Thousand/μl)	116.4 ± 18.9	140.7 ± 35.4	*0.017*
Min. PTT (s)	50.4 ± 13.9	60.6 ± 19.8	0.101
Max. PTT (s)	165 ± 48.7	173 ± 33.9	0.661
Min. PT (s)	13.18 ± 1.33	13.38 ± 1.96	0.737
Max. PT (s)	23.09 ± 7.23	20.24 ± 6.54	0.288
Min. fibrinogen (mg/dl)	115 ± 43.8	175 ± 74.8	*0.009*
Max. fibrinogen (mg/dl)	326 ± 119	336 ± 83.9	0.809
Min. INR	1.14 ± 0.93	1.13 ± 0.13	0.714
Max. INR	1.94 ± 0.67	1.61 ± 1.50	0.171
Platelet V. (ml)	400.90 ± 314.80	434.30 ± 407.19	0.812
FFP V. (ml)	107.60 ± 89.20	86.80 ± 44.15	0.380
Cryoprecipitate V. (ml)	15.45 ± 20.18	7.85 ± 21.5	0.346

**Table 4:** **Arterial blood gas analysis and changes in vital signs**

	**ICH (mean ± SD)**	**No ICH (mean ± SD)**	***p*** **values**
Lowest pH	7.22 ± 0.12	7.24 ± 0.13	0.701
Highest pH	7.48 ± 0.02	7.49 ± 0.06	0.818
Lowest PaCO_2_ (mmHg)	32.73 ± 7.02	30.10 ± 6.6	0.318
Highest PaCO_2_ (mmHg)	65.90 ± 27.9	61 ± 17.5	0.601
Lowest PaO_2_ (mmHg)	52.2 ± 16.7	45.6 ± 16.2	0.298
Highest PaO_2_ (mmHg)	189.7 ± 61.7	179.4 ± 83.9	0.695
Low lactate (mmol/l)	1.143 ± 0.51	1.04 ± 0.59	0.704
High lactate (mmol/l)	5.76 ± 4.41	3.75 ± 3.91	0.335
Min. HR (bpm)	105.6 ± 24.2	101.7 ± 14.5	0.626
Max. HR (bpm)	169.2 ± 20.9	176.9 ± 15.7	0.301
Min. MAP (mmHg)	40.09 ± 6.85	36.52 ± 5.01	0.148
Max. MAP (mmHg)	69.36 ± 4.34	64.19 ± 4.86	*0.006*

## Discussion

Our results showed that low fibrinogen and platelet levels as well as higher MAP were associated with developing ICH. In this report, low fibrinogen level for the first time in the literature was identified as a risk factor for ICH for PPHN patients. Previous studies indicated the risk factors for developing ICH as low platelet counts, difficulty in maintaining platelet counts and ACT levels in normal range with frequent platelet transfusions and frequent adjustments to the heparin drip rate [9-11]. In our study, both groups had similar mean hourly ACT levels as well as heparin infusion doses during the first 12 h on ECMO. Additionally, the maximum ACT and heparin infusion doses per day for the first 5 days were similar. Mean volumes of platelet transfusions between the two groups were not statistically significant (*p* = 0.812).

Significantly, however, lower fibrinogen levels in the ICH group compared to the non-ICH group were noticed (*p* = 0.009). In one study, Kasirajan pointed to lower fibrinogen levels in adult patients with ICH during ECMO; nevertheless, the results did not reach to a statistical significance. In that study, there was a strong correlation between thrombocytopenia and risk of ICH [12]. To our knowledge, this is the first report to show low fibrinogen levels as a risk factor in infants for ICH on ECMO support. Consumption of coagulation factors by the ECMO circuit has been known to occur, and the consequent deposition of fibrin which might cause the transfer of the patient to newly primed circuit has been reported [7,13].

In a previous study, metabolic acidosis, bicarbonate use or inotrope/vasopressor requirement, cardiopulmonary resuscitation, or a left ventricular-assisted device before initiation of extracorporeal life support were identified as risk factors for developing neurological complications on ECMO [14]. In our study, minimum pH levels for both groups were similar during the course of ECMO support and highest lactate levels were not significantly different in both groups. We identified that increased MAP were related to ICH (*p* = 0.006). One can postulate that a lower target MAP may help prevent ICH, as long as an adequate tissue perfusion is maintained.

Systemic arterial hypertension can result in increasing cerebral blood flow in hypoxic newborns if autoregulatory mechanisms decreased or lost. An abnormality in cerebral blood flow is a risk factor for ICH in newborn. One experimental animal study showed that systemic hypertension resulted in germinal matrix hemorrhage [15].

Gestational age was reported to be the most significant predictor of ICH, especially if it is less than 34 weeks [16]. Our study did not support that finding; mean gestational age was 38.1 ± 1.2 weeks in the ICH group vs. 38.3 ± 1.8 weeks for the non-ICH group (Table 1). Hervey-Jumper and colleagues [17] reported that infants less than 30 days of age and requiring ECMO support for cardiac etiology were more prone to have ICH on ECMO. In our study, all patients were less than 30 days of age with a mean age of 2.3 days in both groups at the start of ECMO, and not differing significantly between study groups (*p* = 0.947).

Previously, sepsis as a primary diagnosis was indicated to form an increased risk for ICH in infants compared to non-septic infants, and among septic infants, timing of ICH was reported at less than 72 h on ECMO [18]. In the current study, none of our patients had sepsis, and consequently, sepsis was not a factor for this study.

In our study, the incidence of ICH (34%) is relatively higher compared with ELSO registry data (11%) [8]. Obviously, the one explanation would be the difference in the study group reported here and the ELSO registry patients. One might argue that we report from a subpopulation of the patients undergoing ECMO support procedure and the overall incidence would closely follow the national data.

In recent studies, the search for a better test for monitoring the coagulation status of ECMO patients addressed [19,20]. Anti-Xa test has been indicated as a better correlate for heparin dosing compared to ACT and aPTT tests. Also, antithrombin III test and use of thromboelastography have been mentioned. However, the studies are reported from relatively small group of patients, and further detailed investigations with additional parameters including anti-Xa, antithrombin III, fibrin degradation products, or d-dimers might be useful to establish the utility of these measurements.

One limitation of our study was the small number of patient population with 32 subjects. Furthermore, we reviewed the patient’s data retrospectively. However, it should be pointed out that our data obtained from relatively homogenous group of patients and the study covers a period of 14 years at a high volume reference PICU in a large university medical center, indicating the relative scarcity of patients with similar demographics for this type of analysis. Nevertheless, prospective multi-center studies with a larger patient size might provide a stronger support to the present findings, and the inclusion of larger number of patients on a prospective investigation would eliminate some of the drawbacks that might be resulting from limits on patient number.

## Conclusions

Despite the limitations, our study demonstrated that several factors such as low fibrinogen level, low platelet counts, and high MAP might be associated in development of ICH on ECMO patients. These associations are significant, and some such as low fibrinogen levels are reported for the first time in the literature.

## Abbreviations

ICH: Intracranial hemorrhage
PPHN: Persistent pulmonary hypertension
ECMO: Extra corporeal membrane oxygenation
MAP: Mean arterial pressure
ACT: Activated clotting time
PT: Prothrombin time
aPTT: Activated partial thromboplastin time
INR: International normalized ratio
HUS: Head ultrasound
CT: Computerized tomography
SD: Standard deviation
FFP: Fresh frozen plasma
PVR: Pulmonary vascular resistance
ELSO: Extracorporeal Life Support Organization
